# The Identity of PDGFRA D842V-Mutant Gastrointestinal Stromal Tumors (GIST)

**DOI:** 10.3390/cancers13040705

**Published:** 2021-02-09

**Authors:** Alessandro Rizzo, Maria Abbondanza Pantaleo, Annalisa Astolfi, Valentina Indio, Margherita Nannini

**Affiliations:** 1 Division of Oncology, IRCCS Azienda Ospedaliero-Universitaria di Bologna, 40138 Bologna, Italy; rizzo.alessandro179@gmail.com (A.R.); maria.pantaleo@unibo.it (M.A.P.); 2 Department of Experimental, Diagnostic and Specialized Medicine, University of Bologna, 40138 Bologna, Italy; 3 Department of Translational Medicine, University of Ferrara, 44121 Ferrara, Italy; annalisa.astolfi@unife.it; 4 “Giorgio Prodi” Cancer Research Center, University of Bologna, 40138 Bologna, Italy; valentina.indio2@unibo.it

**Keywords:** gastrointestinal stromal tumor, GIST, PDGFRA, D842V, tyrosine kinase inhibitors

## Abstract

**Simple Summary:**

Among the platelet-derived growth factor receptor (PDGFRA) mutations in gastrointestinal stromal tumors (GIST), the most frequent is the substitution at position 842 in the A-loop of an aspartic acid (D) with a valine (V), widely recognized as D842V, a two-sided mutation providing primary resistance to all currently approved agents for GIST treatment. In recent years, new specific inhibitors have been studied in preclinical and clinical settings, and molecular findings have been accumulated, well describing this complex entity. This paper aims at offering a comprehensive picture of the clinical features and the molecular background of this rare subtype of GIST.

**Abstract:**

The majority of gastrointestinal stromal tumors (GIST) carry a sensitive primary KIT mutation, but approximately 5% to 10% of cases harbor activating mutations of platelet-derived growth factor receptor (PDGFRA), mainly involving the A-loop encoded by exon 18 (~5%), or more rarely the JM domain, encoded by exon 12 (~1%), or the ATP binding domain encoded by exon 14 (<1%). The most frequent mutation is the substitution at position 842 in the A-loop of an aspartic acid (D) with a valine (V) in exon 18, widely recognized as D842V. This mutation, as well known, provides primary resistance to imatinib and sunitinib. Thus, until few years ago, no active drugs were available for this subtype of GIST. Conversely, recent years have witnessed the development of a new specific inhibitor—avapritinib—that has been studied in in vitro and clinical setting with promising results. In light of this primary resistance to conventional therapies, the biological background of D842V-mutant GIST has been deeply investigated to better understand what features characterize this peculiar subset of GIST, and some promising insights have emerged. Hereinafter, we present a comprehensive overview on the clinical features and the molecular background of this rare subtype of GIST.

## 1. Introduction

Gastrointestinal stromal tumors (GIST) account for 0.1–3% of gastrointestinal (GI) malignant tumors and represent the most common mesenchymal malignancy of the GI tract [1]. GIST are worldwide recognized as a milestone of precision oncology, since they represent one of the first examples of oncogene-addicted cancer and a paradigm for genotype-driven targeted therapy. Indeed, with the identification of activating mutations of either KIT or platelet-derived growth factor receptor (PDGFRA) tyrosine kinases as main players in GIST pathogenesis and development, in few years, imatinib has become the backbone for the treatment of unresectable and advanced GIST, whose efficacy is profoundly affected by the underlying tumor genotype [2,3,4,5].

**Figure 1:**
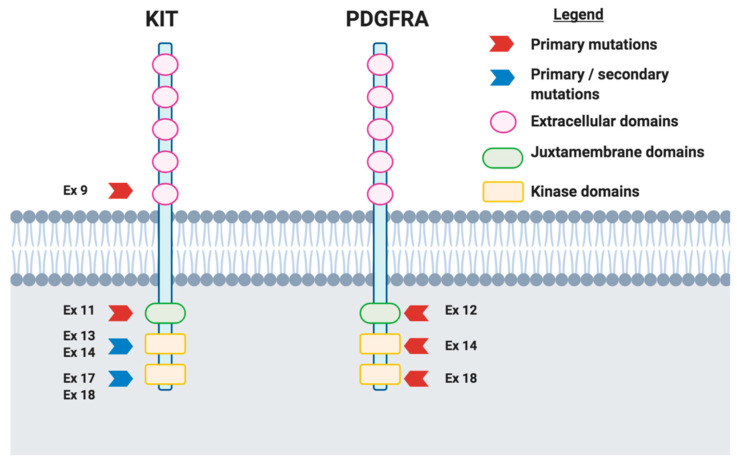
Schematic figure representing primary and secondary KIT and platelet-derived growth factor receptor (PDGFRA) mutations observed in gastrointestinal stromal tumors (GIST). Briefly, exon 11 is the most frequently mutated exon in GIST patients, with these mutations detected in about 60% of cases; exon 9 represents the second most commonly mutated exon (10–12% of cases). Regarding PDGFRA, mutations occurring in a specific hotspot in exon 18 are known as D842V mutations and confer resistance to imatinib.

Although the majority of GIST carry a sensitive primary KIT mutation, about 5% to 10% of cases harbor activating mutations of PDGFRA, mainly involving the A-loop encoded by exon 18 (~5%), or more rarely the JM domain, encoded by exon 12 (~1%), or the ATP binding domain encoded by exon 14 (<1%) (Figure 1) [6]. This rare subset of GIST displays a variable spectrum of sensitivity to imatinib as well as clinical behavior, according to the exon involved and the type of mutation occurred [7]. The most frequent but also intriguing one is the substitution at position 842 in the A-loop of an aspartic acid (D) with a valine (V), widely recognized as D842V, a two-sided mutation. Notably, this mutation provides primary resistance to all currently approved agents due to a conformational change in the kinase domain [7,8,9,10,11]. In addition, few data are available on the clinical activity of agents such as regorafenib in these subpopulations of GIST [12]. Thus, patients with advanced D842V-mutant GIST have always been seen as the “black sheep” of GIST, with a prognosis similar to that of all metastatic GIST in the pre-imatinib era, reporting a median progression-free survival (PFS) of 2.8 months with imatinib and a median overall survival (OS) of 14.7 months [10,11,13].

The fate of this well-defined GIST population has recently changed with the advent of new drugs specifically directed to D842V mutations, such as crenolanib and especially avapritinib [14,15,16]. Furthermore, in recent years, the biological background of D842V-mutant GIST has been deeply investigated to better understand the molecular features of this peculiar subset of GIST, and some promising insights have emerged [17,18,19]. Hereinafter, we present a comprehensive overview on what D842V-mutant GIST have been, what they are now, and what they may be in the near future.

## 2. Clinical–Pathological Identity of D842V-Mutant GIST

Generally, most GIST harboring PDGFRA mutations, including D842V mutants, have a predominant gastric primary localization, a pure or predominant epithelioid morphology, a larger size >5 cm, and low mitotic rate [13,20,21,22,23]. In a study conducted by Agaimy and colleagues where the authors evaluated a combined histomorphological–immunohistochemical pattern analysis of GIST with PDGFRA mutations, PDGFRA-mutant GIST were more frequently epithelioid compared to KIT-mutant GIST (*p* < 0.001) [24]. Of note, these results mirrored previous studies in this setting, where a higher frequency of epithelioid/mixed morphology as well as gastric location were observed in PDGFRA-mutant GIST patients [18,19,24,25]. In addition, a non-statistically significant difference in sex distribution has been observed between cohorts defined by PDGFRA mutations, with a male predominance for exon 18 PDGFRA-mutant GIST.

## 3. TKI Sensitiveness of D842V-Mutant GIST

D842V mutation, as well known, provides primary resistance to all currently approved agents of D842V wild-type disease due to a conformational change in the kinase domain [10,12]. First, this evidence was highlighted in an international, multicenter study conducted by Cassier and colleagues, where the authors explored the role of imatinib in 58 advanced GIST with PDGFRA mutations [10]. Among the included patients, 55% (32/58) had D842V mutations, while 29% presented mutations affecting other codons of exon 18; lastly, mutations in other exons were present in nine GIST patients (16%). According to the results of this report, no responses were observed among 31 evaluable patients with D824V mutation, with 68% (21/31) of this subgroup reporting progressive disease as best response [10]. Notably, the median PFS was 2.8 months (95% confidence interval (CI), 2.6–3.2) for D842V-mutant GIST patients compared to 28.5 months (95% CI, 5.4–51.6) for that of subjects with other PDGFRA mutations (*p* = 0.0001) [10]. Moreover, at a follow-up of 46 months, D842V-mutant GIST patients receiving imatinib displayed a median OS of 14.7 months, while a median OS was not reached for patients without D842V mutations [10].

Similarly, Yoo and colleagues investigated the efficacy of first-line imatinib in 18 GIST patients with PDGFRA mutations [11]. Of note, patients with D842V mutations showed poorer PFS compared to those with non-D842V mutations, with a median PFS of 3.8 months (95% CI, 1.4–6.3) versus 29.5 months (95% CI, 18.3–40.7) (*p* < 0.001), respectively [11]. In addition, poorer OS was observed in the first group, with a median OS of 25.2 months (95% CI, 12.7–37.8) in D842V-mutant GIST and 59.8 months (95% CI, 43.0–76.5) in non-D842V mutant patients (*p* = 0.02) [11].

More recently, the therapeutic landscape of D842V-mutant GIST has seen the advent of avapritinib, a potent and highly selective KIT and PDGFRA type I inhibitor [26]. Indeed, this agent showed for the first time prominent and durable responses in this patient population, never seen before in D842V-mutant GIST [26]. In particular, the antitumor activity of avapritinib has been explored in the recently published NAVIGATOR trial [26]. This multicenter, open-label, phase I trial had a two-part design, including a dose-escalation and a dose-expansion part; eligible patients had GIST that had progressed following imatinib and at least one tyrosine kinase inhibitor (TKI), including sunitinib, regorafenib, sorafenib, dasatinib, pazopanib, or other experimental agents, with similar action. At data cutoff, interesting data in terms of preliminary antitumor efficacy were reported, with 66% (37/56) of D842V-mutant GIST patients remaining on treatment after a median follow-up of 15.9 months [20]. Notably, 88% (49/56) of the PDGFRA D842V-mutant population achieved a response, including 5 cases of complete response (9%) and 44 partial responses (79%) [26].

In addition, an overall manageable safety profile was observed, with 57% of subjects reporting grade 3 or grade 4 treatment-related adverse events, the most frequent being anemia, reported in 17% of cases [26]. Based on these results, in January 2020, the FDA approved avapritinib for the treatment of adults with unresectable or metastatic PDGFRA exon 18-mutant GIST, including PDGFRA D842V mutations [27].

However, as expected, the onset of resistance may occur over time also for D842V-mutant GIST [28]. In fact, a recent study by Bauer et al. provided the first evidence of secondary resistance mutations through sequencing of tumor tissue and plasma biopsies in six out of seven patients receiving avapritinib, suggesting that understanding the emergence of resistance represents an unmet need in this setting [28]. As the authors stated, plasma sequencing may help in identifying resistant clones early, since they arise or are selected during avapritinib treatment. Given that all other escape mechanisms apart from resistance mutations in PDGFRA and its downstream signaling pathways have been found, salvage therapies could be focused on agents inhibiting PDGFRA or on the combination of avapritinib with downstream effectors inhibitors, two therapeutic strategies warranting further preclinical and clinical studies.

Lastly, another type I inhibitor, specifically directed to D842V mutation and other imatinib-resistant PDGFRA kinases, is crenolanib, an agent that is significantly more potent than imatinib according to results of in vitro studies [29]. With these findings, firstly, a phase II trial and then a phase III trial of crenolanib compared to placebo in metastatic GIST with a specific mutation, D842V, were conducted and recently closed, with final results not yet available [30].

## 4. Molecular Identity of D842V-Mutant GIST

D842V-mutant GIST display a very homogeneous molecular profile when compared with KIT-mutant GIST [31,32,33,34]. According to a recent study, with whole-transcriptome analysis, it was observed that D842V-mutant GIST display an overexpression of many genes leading to the activation of G-protein-coupled receptor (GPCR) signaling, including DRD1, SSTR1, and NPBWR1 genes, which could suggest a neural differentiation of D842V tumors [34]. Moreover, an overexpression of quite a large set of chemokines (CCL19, CCL21, CCR2, CCR6, CCR7, and CX3CR1) and prostaglandins (PTGDR, PTGER4, and PTGIR) has been found, which could be a clue regarding a possible role of the tumor microenvironment in this subset of GIST. Conversely, downregulation of several genes belonging to the cell cycle pathway, such as the mini-chromosome maintenance complex genes (MCM family), polymerase genes (POLE, POLD2) and cyclin-dependent kinase (CDK1, CDK2, and CDKN1A), has been observed. Moreover, by looking for fusion transcripts through RNA-sequencing data, no relevant chromosomal aberrations leading to chimeric transcripts have been highlighted, suggesting that D842V tumors may have a rather stable genome. Finally, no actionable molecular events in this population have been reported with whole-exome analysis, confirming that the only recurrent somatic oncogenic mutation was D842V [34]. Many other genes variants were found but were determined to be private genetic events, without pathogenetic significance [34].

Recently, ALK expression was described for the first time in one case of D842V-mutant GIST, without evidence of gene fusion or amplification by FISH and NGS analyses, and the pathogenetic role of this still needs to be clarified [35].

## 5. Immunological Identity of D842V-Mutant GIST

Another unexpected but intriguing novelty of the last year is that PDGFRA-mutant GIST seem to be more immunologically active than other molecular subtypes of GIST [36,37,38].

**Figure 2:**
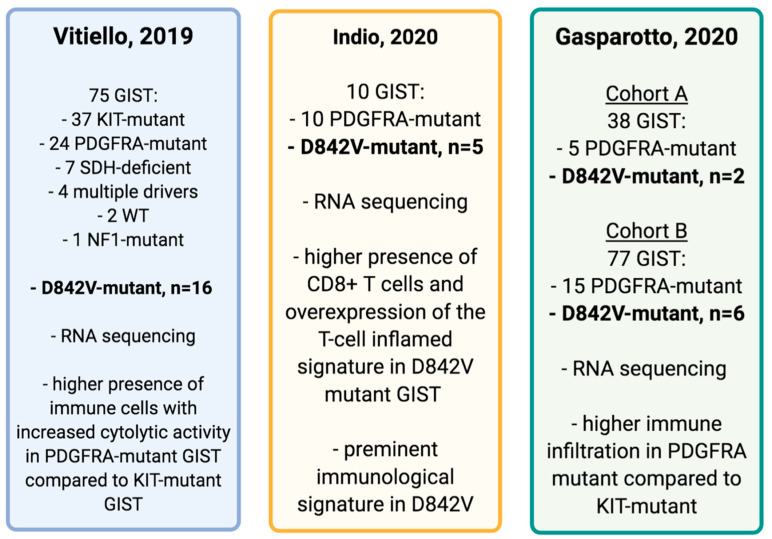
Schematic figure reporting molecular findings of recent immune-profiling studies on GIST, including D842V mutant malignancies [36,37,38]. Abbreviations: GIST: gastrointestinal stromal tumors; n: number; PDGFRA: platelet-derived growth factor receptor A; WT: wild type.

Indeed, assuming that patients with any PDGFRA mutation show a more favorable natural history when compared to those with KIT mutations, Vitiello and colleagues hypothesized that the mutational driver may impact other aspects of tumor biology, specifically, the tumor microenvironment and host immune response (Figure 2). Therefore, by performing RNA-sequencing on 75 surgical specimens from 75 patients with different molecular subset of GIST, they found that PDGFRA-mutant GIST harbor more immune cells than KIT-mutant GIST [37]. In particular, by using single-sample gene set enrichment analyses (ssGSEAs) focused on immune, metabolic, and cell cycle pathways, the authors identified increased immune cell infiltration, greater immune cell activity, and a significant enrichment of immune-related gene sets in PDGFRA-mutant GIST. By immunohistochemical and flow cytometric analyses, Vitiello and colleagues found that PDGFRA-mutant GIST contained more CD45+ and CD8+ cells, with a proportion of immune cells clustered around perivascular structures, a typical feature of adaptive immunity, confirming that PDGFRA-mutant GIST appear to be more immunologically active than KIT-mutant GIST with similar clinicopathologic features. Moreover, differential gene expression analysis revealed that 20 of 93 (21.5%) immune-related genes were significantly differentially regulated between PDGFRA and KIT-mutant GIST, with a significant overexpression of CCR5, BTLA, CD96, CD48, TNFRSF9, TNFSF8, CCR4, CXCL11, CXCR4, KDR, IL6R, TNFRSF8, TNFSF14, TIGIT, TNFRSF17, HLA-DQA2, CXCL14, and CXCL12 in the PDGFRA mutant subgroup. A distinct signaling and cytokine signatures were also found, with a relevant increased expression of CXCL14 in PDGFRA-mutant GIST, which could play a role in the differences in immune infiltration observed between the two subgroups. Finally, the authors found that the D842V mutation produces more high-affinity neoepitopes, binding with many prevalent HLA types, which suggests that the presence of this mutation could be involved in immune responses in a wide variety of patients [37].

Given the known heterogeneity within PDGFRA-mutant GIST, the differential immunological profile of PDGFRA D842V-mutant GIST in comparison with other PDGFRA mutants was subsequently investigated in order to better understand if the previously observed prominent immune features belong to all PDGFRA-mutant GIST or if they represent a specific peculiar fingerprint of the D842V mutant subgroup [36]. Notably, in a subset of 10 samples of untreated primary gastric PDGFRA-mutant GIST, half carrying a D842V mutation and half carrying mutations other than D842V, it was found that the D842V mutant exhibits a significant enrichment of immune- and interferon-related gene signatures and, conversely, a downregulation of several oncogenes, transcription factors, and nuclear receptors [36]. Moreover, the gene expression profiles have also been analyzed with CIBERSORT to evaluate the tumor microenvironment composition, finding a significantly higher abundance of CD8+ T-cells in the D842V patients [36]. The transcriptome profiles were additionally investigated to study the T cell-inflamed signature (TIS) by using an 18-gene signature, composed of IFN-g signaling genes, cytokines, cytotoxic effectors, and antigen-presenting genes. Combining the CIBERSORT results with TIS analysis, a positive correlation was found [36].

Therefore, the marked immunogenicity of PDGFRA-mutant GIST may only belong to the D842V mutant subgroup, and together with the observed lower expression of oncogenes and transcription factors, it could explain the peculiar indolent course of this subset of GIST.

In line with previous data, a recent study confirmed that tumor genotype contributes to shaping the immunogenicity of GIST [32]. In particular, in a cohort of 38 primary, untreated GIST patients, including five cases of PDGFRA-mutant GIST (two D842V mutant) and in a larger cohort of 77 GIST patients, including 15 cases of PDGFRA-mutant GIST (six D842V mutant), PDGFRA-mutant GIST presented a greater extent of immune infiltration and cytolytic activity, which were associated with increased levels of chemokines and a greater number of mutation-derived, high-affinity neoepitopes [38]. To evaluate the potential susceptibility to immunomodulatory-based treatments of the different genotypes, the immunophenoscore (IPS), a machine learning-based classifier based on the expression of HLA genes, immunomodulators, effector, and suppressor cells, capable of predicting the relative sensitivity to immune checkpoint inhibitors (ICIs), was highest in PDGFRA-mutant GIST [38].

Taken together, all of these findings show that there is growing evidence in favor of the marked immunogenicity of PDGFRA-mutant GIST, likely specific of D842V mutants only, providing a possible proof of principle for testing immune-therapeutic approaches in this subset of GIST patients, which remains poor in terms of therapeutic options [39,40]. In particular, several phase I and II clinical trials are evaluating the role of ICIs, as monotherapy or in combination with other anticancer agents, in advanced GIST (Table 1). Results of these studies are highly awaited and will probably clarify if immunotherapeutic agents could enter into the clinical management of GIST patients [41,42].

## 6. Conclusions

D842V-mutant GIST are definitely a rare subgroup of a rare disease, widely recognized as the black sheep of GIST. Even if the fate of this molecularly defined population has recently changed with the advent of avapritinib, treatment options remain very limited, and the problem of how to overcome the expected secondary resistance is now prominent. According to recent reports, salvage therapies based on PDGFRA inhibitors or downstream effectors inhibitors have the potential to overcome acquired resistance to avapritinib, and further efforts are warranted in this direction. Retracing the biological background of D842V-mutant GIST until now recognized, further investigation of their immunological identity seems to represent the most promising step to take in future research.

## Figures and Tables

**Table 1:** Ongoing clinical trials evaluating immunotherapy in GIST.

Clinical Trial	Phase	Treatment	Agents Description
NCT02880020	II	Nivolumab plus ipilimumab	Nivolumab: PD-1 inhibitor Ipilimumab: CTLA-4 inhibitor
NCT02500797	II	Nivolumab plus ipilimumab	Nivolumab: PD-1 inhibitor Ipilimumab: CTLA-4 inhibitor
NCT02834013	II	Nivolumab plus ipilimumab	Nivolumab: PD-1 inhibitor Ipilimumab: CTLA-4 inhibitor
NCT02982486	II	Nivolumab plus ipilimumab	Nivolumab: PD-1 inhibitor Ipilimumab: CTLA-4 inhibitor
NCT01738139	I	Ipilimumab	Ipilimumab: CTLA-4 inhibitor
NCT04000529	I	Spartalizumab	Spartalizumab: PD-1 inhibitor
NCT03609424	I/II	PDR001	PDR001: PD-1 inhibitor
NCT03475953	I/II	Avelumab plus regorafenib	Avelumab: PD-L1 inhibitor Regorafenib: TKI
NCT04258956	II	Avelumab plus axitinib	Avelumab: PD-L1 inhibitor Axitinib: TKI
NCT03291054	II	Pembrolizumab plus epacadostat	Pembrolizumab: PD-1 inhibitor Epacadostat: IDO1 inhibitor
NCT02406781	II	Pembrolizumab plus epacadostat	Pembrolizumab: PD-1 inhibitor Epacadostat: IDO1 inhibitor

Abbreviations: CTLA-4, cytotoxic T-lymphocyte antigen 4; IDO1: indoleamine 2,3-dioxygenase; PD-1, programmed death 1; TKI, tyrosine kinase inhibitor.
